# Systemic path to global health 2050

**DOI:** 10.2471/BLT.25.293951

**Published:** 2026-04-29

**Authors:** Joachim Sturmberg, Maru Mormina, Holger Pfaff, Federica Russo, Mathew Mercuri, Rika Preiser, Valéry Ridde, Peter Delobelle, Garrett Wallace Brown, Fareed Abdullah, Eivind Engebretsen, Kurt C Stange, David Bell, Carmel M Martin, Chris Burman, Felix Tretter, Elisabeth Paul

**Affiliations:** a University of Newcastle, Faculty of Health and Medicine, School of Medicine and Public Health, Callaghan, NSW 2308, Australia.; b European University Institute, Florence, Italy.; c Institute of Medical Sociology, Health Services Research and Rehabilitation Science, Faculty of Medicine and University Hospital Cologne, University of Cologne, Cologne, Germany; d Freudenthal Institute, University of Utrecht, Utrecht, Kingdom of the Netherlands.; e Department of Medicine, McMaster University, Hamilton, Canada.; f Centre for Sustainability Transitions, Stellenbosch University, Stellenbosch, South Africa.; g Université Paris Cité and Université Sorbonne Paris Nord, IRD, Inserm, Ceped, Paris, France.; h Chronic Diseases Initiative for Africa, University of Cape Town, Cape Town, South Africa.; i School of Politics and International Studies, University of Leeds, Leeds, England.; j Office of AIDS and TB Research, South African Medical Research Council, Pretoria, South Africa.; k Sustainable Health Unit, University of Oslo, Oslo, Norway.; l Center for Community Health Integration, Case Western Reserve University, Cleveland, United States of America (USA).; m DB Global Health LLC, Lake Jackson, USA.; n Department of Medicine, Monash University Clayton, Melbourne, Australia.; p Turfloop Graduate School of Leadership, University of Limpopo, Polokwane, South Africa.; q Bertalanffy Center for the Study of Systems Science, Design Forum, Vienna, Austria.; r Research Centre on Health Policies and Systems – International Health, School of Public Health, Université Libre de Bruxelles, Brussels, Belgium.

## Abstract

In 2024, the *Lancet* Commission on Investing in Health proposed targeting investment in 15 priority conditions through 19 modular interventions to improve global health by 2050. While pragmatic, this approach may not fully capture the complex-adaptive nature of health and health systems, nor their social, economic and political determinants. In an iterative, interpretive analysis, proposed global health investment frameworks were mapped against complexity, systems thinking and health epistemology frameworks; five thematic areas were identified for further development: (i) health as emergent from interdependent, social-biological systems; (ii) the non-biomedical determinants driving inequities; (iii) health systems' adaptive requirements; (iv) epistemic injustices that marginalize non-Western perspectives; and (v) the need for context-sensitive, community-led implementation of health measures. Recent major disruptions to international aid financing, while challenging, present a unique opportunity to redesign health investment on more sustainable and locally grounded foundations, where national governments deliberately invest in the social determinants of health as direct health improvement strategies rather than merely as adjacent social policy. To seize this opportunity, we propose five guiding principles for policy-makers: (i) community co-production of interventions; (ii) adaptive governance structures; (iii) complex systems literacy in workforce training to navigate interdependencies and uncertainty; (iv) cross-sectoral partnerships to address determinants of health; and (v) context-sensitive metrics that incorporate community engagement to support learning within health systems. These are not optional enhancements to existing approaches; they are the foundations without which any health investment strategy will continue to treat the symptoms of inequity rather than its causes.

## Résumé

En 2024, la Commission *Lancet* sur l’investissement dans la santé a proposé de cibler les investissements sur 15 pathologies prioritaires à travers 19 interventions modulaires afin d’améliorer la santé mondiale d’ici à 2050. Bien que pragmatique, cette approche ne rend peut-être pas pleinement compte de la nature complexe et adaptative de la santé et des systèmes de santé, ni de leurs déterminants sociaux, économiques et politiques. Dans le cadre d’une analyse itérative et interprétative, les cadres d’investissement proposés en matière de santé mondiale ont été comparés à des cadres de complexité, de pensée systémique et d’épistémologie de la santé. Cette procédure a permis d’identifier cinq domaines thématiques pour un développement plus approfondi: (i) la santé en tant que phénomène émergeant de systèmes sociobiologiques interdépendants; (ii) les déterminants non biomédicaux à l’origine des inégalités; (iii) les exigences d’adaptation des systèmes de santé; (iv) les injustices épistémiques qui marginalisent les perspectives non occidentales; et (v) la nécessité d’une mise en œuvre contextuelle et centrée sur les communautés des mesures sanitaires. Bien qu’elles constituent un défi, les récentes perturbations majeures dans le financement de l’aide internationale offrent une occasion unique de restructurer l’investissement dans la santé sur des bases plus durables et ancrées localement, où les gouvernements nationaux investissent intentionnellement dans les déterminants sociaux de la santé en tant que stratégies directes d’amélioration de la santé plutôt que simplement comme une politique sociale connexe. Pour saisir cette opportunité, nous proposons cinq principes directeurs à l’intention des décideurs politiques: (i) la coproduction communautaire des interventions; (ii) des structures de gouvernance adaptatives; (iii) une formation du personnel pour les amener à maîtriser les systèmes complexes afin de gérer les interdépendances et l’incertitude; (iv) des partenariats intersectoriels pour s’attaquer aux déterminants de la santé; et (v) des indicateurs adaptés au contexte qui intègrent l’engagement communautaire afin de favoriser l’apprentissage au sein des systèmes de santé. Il ne s’agit pas d’améliorations facultatives apportées aux approches existantes, mais des fondements sans lesquels toute stratégie d’investissement dans la santé continuera à traiter les symptômes des inégalités plutôt que leurs causes.

## Resumen

En 2024, la Comisión de *Lancet* sobre Inversión en salud propuso orientar la inversión hacia 15 afecciones prioritarias mediante 19 intervenciones modulares para mejorar la salud mundial de aquí a 2050. Aunque pragmático, este enfoque puede no captar plenamente la naturaleza compleja y adaptativa de la salud y de los sistemas sanitarios, ni sus determinantes sociales, económicos y políticos. En un análisis iterativo e interpretativo, los marcos propuestos de inversión en salud mundial se contrastaron con marcos de complejidad, pensamiento sistémico y epistemología de la salud; se identificaron cinco áreas temáticas para su desarrollo ulterior: (i) la salud como fenómeno emergente de sistemas socio-biológicos interdependientes; (ii) los determinantes no biomédicos que impulsan las inequidades; (iii) las necesidades de adaptación de los sistemas sanitarios; (iv) las injusticias epistémicas que marginan las perspectivas no occidentales; y (v) la necesidad de una implementación de medidas de salud sensible al contexto y liderada por las comunidades. Los recientes cambios significativos en la financiación de la ayuda internacional, aunque plantean desafíos, ofrecen una oportunidad única para rediseñar la inversión en salud sobre bases más sostenibles y con mayor arraigo local, de modo que los gobiernos nacionales inviertan deliberadamente en los determinantes sociales como estrategias directas de mejora de la salud, en lugar de tratarlos como meras políticas sociales complementarias. Para aprovechar esta oportunidad, se proponen cinco principios rectores para los responsables de la formulación de políticas: (i) la coproducción comunitaria de intervenciones; (ii) las estructuras de gobernanza adaptativas; (iii) la alfabetización en sistemas complejos en la formación del personal para abordar interdependencias e incertidumbre; (iv) las alianzas intersectoriales para abordar los determinantes de la salud; y (v) las métricas sensibles al contexto que incorporen la participación comunitaria para apoyar el aprendizaje dentro de los sistemas sanitarios. Estas medidas no son mejoras opcionales de los enfoques existentes; constituyen la base necesaria para que cualquier estrategia de inversión en salud deje de centrarse en los síntomas de la inequidad y aborde sus causas.

## ملخص

في عام 2024، قامت لجنة *Lancet * للاستثمار في الصحة، باقتراح توجيه الاستثمار نحو 15 حالة ذات أولوية من خلال 19 تدخلاً معيارياً لتحسين الصحة العالمية بحلول عام 2050. ورغم أن هذا الأسلوب يعد عمليًا، إلا أنه قد لا يُغطي بشكل كامل الطبيعة المعقدة والمتغيرة للصحة والنظم الصحية، ولا محدداتها الاجتماعية والاقتصادية والسياسية. وفي تحليل تفسيري تكراري، تم الربط بين أطر عمل الاستثمار المقترحة في الصحة العالمية، وأطر عمل التعقيد والتفكير النظمي ونظرية المعرفة الصحية؛ وتم تحديد خمسة مجالات موضوعية لمزيد من التطوير: (1) الصحة كنتيجة لأنظمة اجتماعية بيولوجية مترابطة؛ و(2) المحددات غير الطبية الحيوية التي تؤدي إلى عدم المساواة؛ و(3) متطلبات التكيف لنظم الصحة؛ و(4) المشكلات المعرفية التي تقلل من أهمية وجهات النظر غير الغربية؛ و(5) الحاجة إلى تنفيذ الإجراءات الصحية بطريقة تراعي الأوضاع وبقيادة المجتمع. الاضطرابات الكبيرة مؤخرًا في تمويل المساعدات الدولية، رغم أنها تمثل تحديًا، إلا أنها تمثل فرصة فريدة لإعادة تصميم الاستثمار في الصحة على أسس أكثر استدامة وتأصلاً في الوضع المحلي، حيث تستثمر الحكومات الوطنية بشكل متعمد في المحددات الاجتماعية للصحة كاستراتيجيات مباشرة لتحسين الصحة، بدلًا من اعتبارها مجرد سياسة اجتماعية مجاورة. ولانتهاز هذه الفرصة، فإننا نقترح خمسة مبادئ توجيهية لواضعي السياسات: (1) المشاركة المجتمعية في وضع التدخلات؛ و(2) هياكل حوكمة قابلة للتكيف؛ و(3) الإلمام بالأنظمة المعقدة في تدريب القوى العاملة للتعامل مع الترابطات وعدم اليقين؛ و(4) الشراكات بين القطاعات لمعالجة محددات الصحة؛ و(5) مقاييس حسب الأوضاع تدمج المشاركة المجتمعية لدعم التعلم داخل النظم الصحية. هذه ليست تحسينات اختيارية للأساليب المتواجدة، بل هي الأسس التي دونها ستستمر أي استراتيجية استثمار في الصحة في معالجة أعراض عدم المساواة بدلًا من أسبابها.

## 摘要

2024 年，《柳叶刀》健康投资委员会提出通过 19 项模块化干预措施，对 15 类重点疾病进行针对性投资，以提高 2050 年全球的健康状况。该方法虽然务实，但是可能无法充分体现健康和健康系统复杂且自适应的特性，也无法把握其社会、经济和政治决定因素。在一项迭代式、阐释性分析中，我们将所提议的全球健康投资框架与复杂性理论、系统思维及健康认识论框架进行了比较，确定了五大有待进一步发展的主题领域：(i) 健康是从相互依存的社会—生物系统而涌现产生；(ii) 非生物医学导致不公平现象；(iii) 卫生系统适应性要求；(iv) 将非西方视角边缘化的认识论不公正；以及 (v) 需要贴合本土情景、社区主导的健康措施实施方式。近期对国际援助资金的重大扰动极具挑战，但也带来独特的机会，得以在更可持续、更贴合本土实际的基础上，重新规划全球健康投资，各国政府也有意识地主动投资健康社会决定因素，以作为直接核心策略，而非只将其当作附属的社会政策。为了抓住这一机遇，我们为政策制定者提出五项指导原则：(i) 社区共同创建干预举措；(ii) 适应性治理架构；(iii) 在从业人员培训中融入复杂系统素养，以应对各种相互依赖与不确定性；(iv) 建立跨部门协作机制，以处理健康决定因素；(v) 制定适合本土情况的指标，纳入社区参与维度，支持卫生系统内部的学习。这些不是对现有工作方法的可选性升级补充；它们是不可或缺的底层支撑，少了它们，任何健康投资策略都将继续缓解健康不公的表面问题，而非解决其根本诱因。

## Резюме

В 2024 году комиссия журнала *Lancet* по инвестициям в здравоохранение представила план по улучшению глобального здоровья к 2050 году, который включает целевые инвестиции в борьбу с 15 приоритетными заболеваниями путем 19 модульных вмешательств. Несмотря на прагматичность такого подхода, в нем могут не учитываться в полной мере сложность и адаптивный характер здоровья и систем здравоохранения, а также их социальные, экономические и политические детерминанты. В ходе итерационного интерпретационного анализа предложенные концепции инвестирования в глобальное здравоохранение были сопоставлены с концепциями теории сложности, системного мышления и эпистемиологии здоровья, при этом было выявлено пять тематических областей дальнейшего развития: (i) здоровье как явление, возникающее во взаимозависимых социобиологических системах; (ii) детерминанты небиомедицинского характера, приводящие к неравенству; (iii) требования к адаптивности систем здравоохранения; (iv) эпистемическая несправедливость, маргинализирующая точки зрения, отличные от западной; (v) необходимость внедрения мер в сфере здравоохранения с учетом местных условий и при активном участии самих сообществ. Недавние серьезные случаи прекращения международного финансирования помощи хотя и создают трудности, но открывают уникальную возможность перевести инвестиции в здравоохранение на более устойчивую основу с опорой на местные ресурсы, при которой национальные правительства будут целенаправленно инвестировать в социальные детерминанты здоровья как в прямой способ улучшения здоровья, а не только рассматривать их как смежное направление социальной политики. Чтобы воспользоваться такой возможностью, авторы предлагают пять руководящих принципов для лиц, принимающих политические решения: (i) совместную разработку и реализацию мер с участием местных сообществ; (ii) адаптивные структуры управления; (iii) подготовку кадров с акцентом на понимание сложных систем, чтобы они могли действовать в условиях неопределенности и учитывать взаимозависимость; (iv) межсекторальное партнерство при работе с факторами, влияющими на здоровье; (v) контекстно зависимые показатели, которые учитывают вовлечение сообществ для поддержки процесса обучения в рамках систем здравоохранения. Это не факультативные улучшения уже существующих подходов, а фундамент, без которого любая стратегия инвестиций в здравоохранение будет и впредь пытаться исправлять симптомы неравенства, а не их причины.

## Introduction

As global health agendas look beyond the United Nations’ *Transforming our world: the 2030 agenda for sustainable development*,^1^ it is clear that linear, disease-specific, health-care strategies (for example, single-disease or step-by-step approaches) remain dominant despite decades of evidence about the social and systemic determinants of health. For example, the *Lancet* Commission on Investing in Health report *Global health 2050: the path to halving premature death by mid-century*^2^ proposes that, with substantial investment, global health could markedly improve by mid-century through a targeted approach that focuses on 15 priority conditions, namely eight infectious and maternal health conditions and seven noncommunicable diseases, packaged into 19 intervention modules.

Despite efforts to improve health-care services and delivery, such as those proposed by the *Lancet* Commission, challenges remain in realizing benefits for communities. Achieving health outcomes will require a broader framing that reflects the complex-adaptive nature of health systems. Table 1 summarizes the core distinctions between a disease-focused approach, which underpins many current global health investment frameworks, and a health systems-focused approach that is grounded in systems thinking and takes complexity into account. These approaches are not mutually exclusive traditions but understanding their complementarity and differences helps clarify what a more comprehensive, systemic, global health strategy needs to address.

**Table 1:** Contrasting disease-focused and health systems-focused approaches to global health improvement

Dimension	Disease-focused approach^a^	Health systems-focused approach^a^
Unit of analysis	Individual diseases or conditions, which are addressed through targeted programmes	The whole person embedded in social, economic and environmental systems
Model of causality	Linear: specific pathogens, risk factors or genetic determinants cause specific diseases	Multicausal and emergent: health outcomes arise from interacting biological, social and structural factors
Knowledge and evidence	Biomedical and epidemiological expertise, with an emphasis on randomized trial evidence, standardized protocols and modelling studies	Pluralistic: integrates knowledge from the biomedical and social sciences and from the community and lived experience
Intervention logic	Standardized, modular interventions assumed to have context-independent efficacy	Adaptive, context-sensitive interventions co-designed with communities to fit local conditions
Health system role	A delivery platform for disease-specific programmes, with system strengthening as a by-product	A core object of investment, though resilient, people-centred systems are prerequisites for all other health gains
Financing	Mostly externally co-funded, vertical programmes that target specific diseases or conditions	Sustainable domestic financing that deliberately invests in the social determinants of health, such as education, housing, employment and the environment, as direct health improvement strategies, not merely as adjacent social policy
Governance	Top-down: global priorities are translated into national programmes with defined targets	Adaptive and distributed: local constituencies co-design pathways within broad goals, thereby enabling iterative learning
Metrics of success	Disease-specific targets, such as mortality rates, disease incidence or intervention coverage	System-level and community-defined outcomes, including well-being, equity, sustainability and resilience

^a^ The columns represent intellectual traditions that inform health policy but, in practice, effective strategies must draw on both.

The recommendations of the *Lancet* Commission on Investing in Health and, more generally, work emanating from the World Bank’s Disease Control Priorities approach,^3^ are built on a technical view of health and disease based on modular solutions, which may underestimate the social, economic and political determinants of most preventable premature death. This approach focuses on biomedical interventions that target specific diseases or conditions rather than sustaining a high-quality health system capable of delivering people-centred primary care that is optimized to the local socioeconomic context.^4^ Similar objections have also been raised elsewhere.^5^

In this paper, we approach the goal of improving global health from a foundation that is both epistemological (that is, how we know and understand health) and normative, in that it makes claims about how health systems ought to be designed and governed. First, we lay the foundations by examining the concepts of health and disease through a systems lens. We then explore the limitations of disease-focused thinking and the practical pathways towards a more systemic alternative, focusing on the dynamic interconnectedness and interdependencies between the biological, social, economic and environmental factors that shape health outcomes. To do so, we draw on iterative, interpretive analysis to map current global health investment thinking against established scholarship on complexity, systems and health epistemology. Five thematic areas emerged through this process via a consensus-based synthesis: (i) adopting a systems perspective; (ii) going beyond linear technical solutions; (iii) going beyond modular approaches; (iv) going beyond top-down, normative approaches; and (v) adopting a complex-adaptive systems approach.

## A systems perspective

The World Health Organization (WHO) defines health as, “a state of complete physical, mental and social well-being and not merely the absence of disease or infirmity.”^6^ This definition moves beyond a purely biomedical perspective by incorporating social well-being, thereby suggesting that health outcomes are not determined only by well-defined physical diseases but are also shaped by social, economic and environmental conditions.

Decades of literature on the social determinants of health have shown how health outcomes arise from dynamic interactions between interconnected and interdependent individual biological, social, commercial and environmental factors.^4^^,^^7^^–^^11^ Traditional biomedical models are not designed to capture these interdependencies but instead rely on linear causality, where specific pathogens or genetic factors are seen as the primary causes of disease. Yet, factors such as income, education, housing, employment, health literacy and access to health care interact in complex and dynamic ways to shape an individual’s health.^12^^–^^16^ For example, poor access to healthy foods and limited physical activity, both influenced by poverty and education, increase the risk of developing conditions such as obesity and diabetes, which in turn increase the risk of communicable and other noncommunicable diseases alike.^17^

Understanding these dynamics requires a complex-adaptive systems approach, underpinned by systems and complexity thinking.^18^ Systems thinking allows us to view health as the product of interdependent networks, where feedback loops, emergent properties and adaptive responses shape outcomes, thereby moving beyond the explanatory limits of linear, single-cause-and-effect models.^19^^,^^20^ Complexity thinking recognizes that individual health journeys, health systems and disease processes exhibit nonlinearity, self-organization and unpredictability.^21^ Small changes in one part of a system can produce disproportionate effects on population health and equity, as starkly illustrated by the coronavirus disease 2019 pandemic, in which a single zoonotic spillover event cascaded into global health, economic and social disruption that no linear model could have predicted or contained.^22^^,^^23^ This complexity-infused framing allows for the design of interventions that are adaptive, context-sensitive and resilient in the face of the uncertainty inherent in nonlinear dynamics.^24^

## Beyond linear technical solutions

The importance of treating and preventing diseases, especially infectious diseases and maternal and neonatal conditions, is beyond question. Yet, focusing primarily on biomedical interventions within a modular approach risks overlooking both the dynamic interdependencies between biological, social, economic and environmental factors, and the synergistic effects the political, economic and social root causes of a disease have on its symptoms.^25^

The experience of countries with well-developed health systems illustrates this clearly. The National Health Service in the United Kingdom of Great Britain and Northern Ireland guarantees universal health coverage, yet there are huge differences in life expectancy between richer and poorer regions, and the variation in average mortality between regions is higher than in comparable countries with more holistic approaches to health care.^26^ Socioeconomic inequalities reflected in differential access to housing, employment and social capital consistently predict shorter life expectancy, regardless of health care provision.^26^^,^^27^ A health system that treats disease but leaves its social roots intact is not eliminating a problem, instead, it is perpetuating the conditions that sustain it.^28^

Addressing this omission requires deliberate attention to the non-medical causes of poor health that governments have both the power and the duty to act on, including extreme poverty, low educational attainment, environmental degradation and the influence of unhealthy commodity industries in, for example, the food, tobacco and alcohol sectors.^7^^,^^29^ A whole-of-system approach that incorporates the political, economic and social determinants of health does more than improve health outcomes; it enhances political accountability, fosters intersectoral collaboration across education, housing, transportation and many other sectors, and builds the kind of resilient social infrastructure that no disease-specific programme alone can create.^30^

## Beyond modular approaches

Addressing the social roots of ill health, as argued above, requires more than redirecting political will. Such an approach depends on health systems designed to operate as complex-adaptive systems rather than as delivery platforms for discrete interventions. Yet, even within its own terms, a modular approach to delivering interventions faces a fundamental challenge: biomedical interventions do not have an intrinsic efficacy that is independent of the context and system delivering them. Vaccination, for example, among the simplest and most cost-effective of public health tools, can be substantially less effective in the real world than indicated by its efficacy in clinical trials.^31^ There can be significant variations related to the context in which it is implemented, even within the same country.^32^ Every intervention, from the simplest to the most complex, both shapes and is shaped by the system within which it operates.

These complexities point to the centrality of health system strengthening, which the *Lancet* Commission on Investing in Health’s report appears to consider simply as a by-product of investing in priority disease programmes, rather than as the foundational condition that makes all other health gains possible. Health systems are complex-adaptive systems in their own right and exhibit the same nonlinearity, emergent properties and context-dependence that characterize the health challenges they are designed to address.^33^

For people living with multiple chronic conditions, this is not an abstract point: care organized around individual diseases frequently produces iatrogenic harm, as interventions optimized for one condition interact in ways that are difficult to anticipate when the system is not configured to view care holistically. Care that attends to the person as a whole and explicitly considers tipping points, feedback effects and the central importance of social and environmental context consistently produces better outcomes.^34^

Health system strengthening must therefore be multifaceted and context-sensitive; it should encompass governance, financing, workforce and infrastructure and be oriented around a clearly defined, systemic purpose rather than a collection of programme targets. As Hazlitt observed in 1946, “The art of economics consists in looking not merely at the immediate but at the longer effects of any act or policy; it consists in tracing the consequences of that policy not merely for one group but for all groups.”^35^ This principle applies with full force to health system investment. Critically, local constituencies and communities should be empowered to co-design the pathways through which that systemic purpose is achieved, thereby generating the kind of bottom-up adaptability that no top-down framework can replicate.^36^ This approach is not merely a design preference, but rather a practical necessity. As national governments in low- and middle-income countries face the urgent challenge of replacing disrupted international aid with sustainable domestic and cross-sectoral financing, health systems with genuine adaptive capacity, deep community roots and governance structures that span the social determinants of health are better positioned to meet that challenge.^37^^,^^38^

## Beyond top-down approaches

Building the kind of adaptive, community-rooted health systems described above involves more than structural reform; it requires a fundamental rethinking of who defines the purpose and priorities of those systems in the first place. *The world health report 2008: primary health care now more than ever* distinguished three models of health-care provision:^39^ (i) conventional ambulatory medical care in clinics or outpatient departments, focusing on illness and cure in the context of episodic curative care; (ii) disease control programmes, focusing on priority diseases and related programme-defined, disease control interventions; and (iii) people-centred primary care, focusing on communities’ health needs, with people considered as partners in managing their own health and that of their community. The first two models position communities as recipients of care; only the third positions them as its co-producers. This third model is, therefore, best placed to address health holistically, including its underlying non-medical determinants, and it is towards this model that a systems approach naturally points.

Systems thinking is inherently normative in this regard: it does not simply describe the interconnections between health, disease and the environment, but places personal, social, cultural and ethical considerations at the core of any account of health and, therefore, at the core of intervention design.^40^ This normative orientation matters because perceptions of health and illness are not uniform, instead they are shaped by personal experiences and by historical, social and environmental contexts that cannot be read off from biomedical indicators alone.^20^Improving health therefore requires genuine receptiveness to the voices of those living with illness and those maintaining health, as well as to the broader societal norms and values within which both are experienced. A systems approach treats communities not as intervention targets but as active participants and coproducers of their own health.^41^

Co-producing knowledge about health, however, demands first confronting whose knowledge has historically counted. Epistemic injustice arises when a lack of credibility or interpretive deficits limit certain groups’ ability to contribute to knowledge, and when exclusion or distortion of that knowledge perpetuates inequities in global health, particularly in a context where Western biomedical frameworks have historically dominated while non-Western perspectives have been marginalized.^42^^,^^43^ Most world-views, from traditional Chinese medicine and Indian Ayurveda to African, Islamic and various Indigenous perspectives, view health as a holistic concept inseparable from community, culture and the natural environment,^20^^,^^44^^–^^46^ which reflects a systems perspective.^20^ These are not prescientific beliefs to be superseded; they are sophisticated epistemological frameworks that capture dimensions of health and well-being that biomedical models are not designed to see. In many communities that are the recipients of health interventions, these frameworks are, more importantly, the dominant ways of understanding illness, healing and well-being. Epistemically just coproduction of knowledge about health that integrates diverse scientific and cultural knowledge and lived experiences on an equal basis is, therefore, not merely an ethical obligation but also a practical necessity for designing interventions that are effective across the full diversity of human contexts.^47^

## Adopting a systems approach

In the preceding sections we have argued that health outcomes are shaped by dynamic, interdependent systems; that their biological, social, economic and environmental determinants demand deliberate cross-sectoral investment; that health systems must be strengthened as complex-adaptive entities in their own right; and that the communities they serve must be genuine coauthors of their design and purpose. The practical question is what this looks like when translated into action.

The answer, crucially, is that it looks different everywhere and that this is not a weakness but is the defining feature of a systems approach. Successful community-focused, primary care systems clearly demonstrate this feature. Two examples are the Nuka System of Care in Alaska,^48^ developed with and for Alaska Native and American Indian communities, and South Africa’s community-oriented, primary care model,^49^ built around the specific social and epidemiological realities of post-apartheid urban health. Although these systems share no single blueprint, both have achieved sustained improvements in well-being and longevity precisely because they were designed from the ground up rather than delivered from the top down. Responsiveness to changing needs and circumstances enables the kind of ongoing adaptation that produces dynamic stability in a complex-adaptive system, not the stability of a fixed structure, but the stability of a living system that continues to function because it continues to learn.

A persistent assumption among many proposals on how to meet global health needs is that health improvement in low- and middle-income countries is a problem to be solved by external experts and technocrats who usually adopt a so-called foreign gaze.^50^ This approach is not merely a strategic error, it is itself a social determinant of the inequities it purports to address.

Table 2 distils the key principles of a complex-adaptive system approach and illustrates how they were operationalized in the Nuka System of Care. The lesson for health system leaders is not that the specific components of the Nuka System should be replicated. Doing so would fall back on a one-size-fits-all logic this paper argues against. What should be replicated is the more abstract process by which the health system was built: beginning with the community, working outward to the system, and remaining continuously responsive to both.

**Table 2:** The principles of systems and complexity thinking approaches to health system improvement, their application and the example of the Nuka System of Care in Alaska, United States of America

Principles	Application for improving global health	Translation of these principles in the redesign of health-care implementation in the Nuka System of Care^48^
Adopt a complex-adaptive systems approach to address the interconnected, systemic drivers of health and disease	Engage all community stakeholders in deciding on health system priorities for their unique context	Community members were engaged in defining the causes of poor health and inadequate health care, which enabled them to co-design the solutions best adapted to local issues
Strengthen health systems by investing in governance, infrastructure and capacity to build resilient and adaptive systems	Build a publicly funded, comprehensive primary care system as the basis for achieving the goals of Global Health 2050 report^2^	Leadership focused both on ensuring services were adequately resourced to deliver identified health-care needs and on evaluating the processes of care delivery adopted and their health outcomes
Adopt a people-centred, primary care approach that engages communities as active participants in their health and health care, including building a local health-care system best adapted to their health and cultural needs	Engage all community stakeholders in deciding on health system priorities for their unique context	Community members were asked about their ideal health system and identified three system requirements: (i) a personal relationship with their primary care provider; (ii) being treated with courtesy, respect and cultural understanding; and (iii) having access to care when needed
Promote epistemic justice and inclusiveness to integrate knowledge from diverse scientific fields and lived experience into health-care delivery and health system design	Increase social investment and decrease socioeconomic gradients, which are the drivers of the 15 priority conditions identified by the *Lancet *Commission on Investing in Health^2^	NA
Shift the focus from disease management to health promotion centred on the social determinants of health and the creation of healthy environments	Focus on the stressors that lead to unhealthy health behaviours and premature morbidity and mortality	The Charter of the Southcentral Foundation was amended to include the goals of: (i) improving the health and social conditions of Alaska Native people; and (ii) enhancing local culture and empowering individuals and families to take charge of their lives^48^
Foster intersectoral collaboration to address the social determinants of health, such as education, housing, community facilities and public transportation	Develop local, regional and national primary care networks to: (i) identity gaps in health and social care services; and (ii) provide bottom-up feedback to facilitate cross-sectoral integration of health, social and community needs	NA
Embrace the complexities inherent in tasks because they allow for the emergence of flexible, context-sensitive solutions to the unpredictable and dynamic nature of health systems	Engage all community stakeholders in deciding on health system priorities for their unique context	Ongoing community consultations help identify changing and newly emerging health needs; and ongoing feedback provides the community with information on service changes and their impacts on care delivery and health outcomes

NA: not applicable.

## Conclusion

In a moment when declining international aid is forcing national governments, especially those in low- and middle-income countries, to reimagine how health systems are financed and governed, what is widely experienced as a crisis process may also prove to be one of the most important catalysts for health system transformation in a generation. This disruption aligns with long-standing calls for greater national ownership and community-driven priorities. For decades, external funding has shaped health priorities in low- and middle-income countries around the disease-specific agendas of donors rather than the systemic needs of communities. The withdrawal of that funding, painful as it is in the short-term, opens space for a fundamentally different approach. In this approach, national governments, communities and health-system leaders define for themselves what their health systems are for and how they should be financed, while considering the local burden of disease and epidemiological trends. Processes such as community co-production, adaptive governance, cross-sectoral investment in the social determinants of health and context-sensitive metrics offer not just a more equitable path to Global Health 2050,^2^ but arguably the only sustainable one. 

That approach, as we argue in this paper, must be systemic. Priority disease-focused investments save lives and must continue but need to consider the conditions that would make them more effective, more equitable and more sustainable, which we argue would amount to creating interventions that resonate with adaptive health systems, cross-sectoral financing, community coproduction and epistemically just governance. These are not optional enhancements to a biomedical programme; they are the foundations without which any programme, however well designed, will continue to treat the symptoms of inequity rather than its causes.

To seize this moment, we propose five guiding principles for policy-makers and health system leaders: (i) invest in community co-production of health interventions, thereby ensuring that local stakeholders actively shape priorities and solutions; (ii) build adaptive governance structures that enable continuous policy learning and responsiveness to emerging needs; (iii) embed complex systems literacy into health workforce training to navigate interdependencies, nonlinearity and uncertainty; (iv) strengthen cross-sectoral partnerships to systematically address the social, commercial and environmental determinants of health; and (v) prioritize context-sensitive metrics that incorporate community engagement and support learning health systems, rather than consider only universal targets. These principles are not a blueprint, as blueprints are precisely what a systems approach resists. They are navigational commitments, namely orientations that keep health systems pointed towards equity, adaptability and community ownership as the landscape around them changes. The persistent assumption that health is primarily a biomedical problem, which is solvable through the right combination of interventions delivered at scale, has not failed for lack of trying. The assumption has failed because it was never an accurate description of what health is. Replacing it with frameworks, financing and governance structures that reflect the full complexity of human health in its social, environmental and cultural context is not merely a scientific imperative, it is a moral one.
